# Ion tracks in silicon formed by much lower energy deposition than the track formation threshold

**DOI:** 10.1038/s41598-020-80360-8

**Published:** 2021-01-08

**Authors:** H. Amekura, M. Toulemonde, K. Narumi, R. Li, A. Chiba, Y. Hirano, K. Yamada, S. Yamamoto, N. Ishikawa, N. Okubo, Y. Saitoh

**Affiliations:** 1 National Institute for Materials Science (NIMS), Tsukuba, Japan; 2 CIMAP, Caen, France; 3 National Institutes for Quantum and Radiological Science and Technology (QST), Takasaki, Japan; 4 Shandong University, Jinan, China; 5 Japan Atomic Energy Agency, Tokai, Japan

**Keywords:** Atomic and molecular collision processes, Surface patterning

## Abstract

Damaged regions of cylindrical shapes called ion tracks, typically in nano-meters wide and tens micro-meters long, are formed along the ion trajectories in many insulators, when high energy ions in the electronic stopping regime are injected. In most cases, the ion tracks were assumed as consequences of dense electronic energy deposition from the high energy ions, except some cases where the synergy effect with the nuclear energy deposition plays an important role. In crystalline Si (c-Si), no tracks have been observed with any monomer ions up to GeV. Tracks are formed in c-Si under 40 MeV fullerene (C_60_) cluster ion irradiation, which provides much higher energy deposition than monomer ions. The track diameter decreases with decreasing the ion energy until they disappear at an extrapolated value of ~ 17 MeV. However, here we report the track formation of 10 nm in diameter under C_60_ ion irradiation of 6 MeV, i.e., much lower than the extrapolated threshold. The diameters of 10 nm were comparable to those under 40 MeV C_60_ irradiation. Furthermore, the tracks formed by 6 MeV C_60_ irradiation consisted of damaged crystalline, while those formed by 40 MeV C_60_ irradiation were amorphous. The track formation was observed down to 1 MeV and probably lower with decreasing the track diameters. The track lengths were much shorter than those expected from the drop of *S*_e_ below the threshold. These track formations at such low energies cannot be explained by the conventional purely electronic energy deposition mechanism, indicating another origin, e.g., the synergy effect between the electronic and nuclear energy depositions, or dual transitions of transient melting and boiling.

## Introduction

High energy ion irradiation often introduces radiation-induced damage (RID) in materials, which normally decreases with decreasing incident ion energy *E*. However, the present study reports that ion tracks, i.e., a type of RID, in crystalline silicon (c-Si) disappear and re-appear (or at least, decrease and increase) with monotonically decreasing *E*. High energy ions in the electronic energy loss regime are called swift heavy ions (SHIs)^1,2^. The SHIs penetrate materials with almost straight trajectories, along which massive amount of energy is deposited, resulting in cylindrical shaped damage regions known as ‘latent ion tracks’. The first observation of such tracks was in 1958 in lithium fluoride^3^, where the tracks were attributed to naturally occurring nuclear fissions of radioactive impurities. Since the 1980s, the study of the tracks rapidly advanced with use of SHIs from large accelerators being applicable. Experiments employing up to GeV-level U ions have shown that no SHIs create ion tracks in c-Si^4,5^.

With increasing ion energy *E*, the electronic energy deposition (*S*_e_) in materials increases proportional to *E*^1/2^ in the Lindhard-Scharff (low energy) regime^6^, but decreases in the Bethe-Bloch (high energy) regime^6^, with passing the Bloch peak. No greater deposition at the Bloch peak is possible for a given pair of ion species and material. Crystalline silicon (c-Si) is one of the most important materials in today’s technology. Even using ^238^U ions (i.e., the heaviest quasi-stable ions), the maximum *S*_e_ at the Bragg peak only reaches ~ 25 keV/nm in c-Si, which is lower than the electronic deposition threshold of ~ 30 keV/nm, which was determined from the cluster ion experiments later.

The high threshold of c-Si was overcome by utilizing fullerene (C_60_) ions. Since, in this case, all sixty C atoms are injected in almost the same position at almost the same time, roughly sixty times greater energy deposition than the monomer can be achieved. In the late 1980s, Canut et al.^7^ and Dunlop et al.^8^ simultaneously succeeded in producing latent ion tracks in c-Si by using 40 and 30 MeV C_60_ ions in the Orsay facility in France. As the C_60_ energy decreases from 40 MeV, 30 MeV, to 25 MeV, the track mean diameters decreased from 10.5 nm, 8.4 nm, to 6 nm. An extrapolation suggested that the tracks would no longer be formed at less than 17 MeV^7^.

In this letter, we report the ion track formation in c-Si induced by C_60_^+^ ion irradiation between 1 and 6 MeV, which are much lower than the above-mentioned threshold of 17 MeV. Surprisingly, large tracks of ~ 10 nm in diameter were formed with 6 MeV irradiation, which are comparable in size to those with 40 MeV irradiation. Furthermore, although the sizes decreased, the tracks were observed down to 1 MeV C_60_ irradiation or lower. These observations clearly indicate that the tracks are formed at much lower energy than the threshold of 17 MeV which was extrapolated from the high energy data, indicating another mechanism for the track formation which is active under 1–6 MeV C_60_ irradiation. Possible candidates are synergy effects between the electronic and the nuclear energy deposition (*S*_e_ and *S*_n_)^9–12, ^ and the transient boiling-melting transitions with recrystallization.

## Results

**Figure 1:**
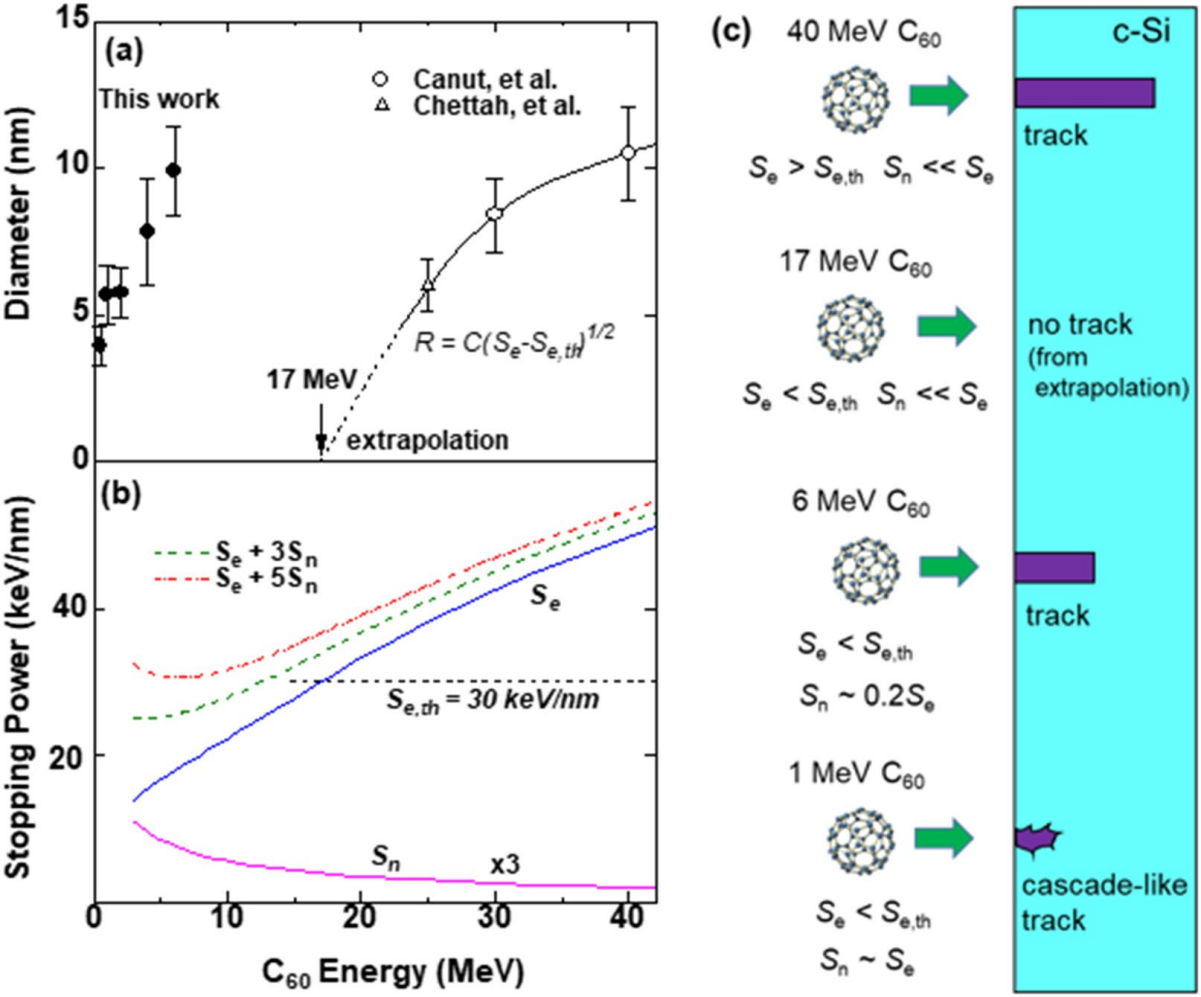
Ion energy dependence of (**a**) track diameter, (**b**) electronic (*S*_e_), nuclear (*S*_n_), and effective (*S*_eff_) stopping powers (*K* = 3 and 5). In (**a**), the open circles, open triangle, and closed circles, respectively, denote data from Canut et al.^4^, Chettah et al.^7^, and the present study. *S*_e,th_ denotes the threshold *S*_e_ extrapolated from the data at 25 MeV and higher. (**c**) Schematic depiction of the ion energy dependence of track formation and its relationship to the stopping powers.

The open symbols in Fig. 1a show the ion track diameters in c-Si, formed by C_60_ ion irradiation (*E* ≥ 25 MeV), as reported in past literature^7,8,13^. The energy dependencies of the electronic and nuclear stopping powers, *S*_e_ and *S*_n_, of C_60_ ions in c-Si are plotted in Fig. 1b, which were approximated as the sum of sixty independent carbon monomer ions with the energy of (*E*/60) each, and expressed as,

1 $$S_{i} (E,{\text{ C}}_{{{6}0}} ) = {6}0S_{i} (E/{6}0,{\text{ C}}_{{1}} )$$

where *i* = *n* (nuclear) or *e* (electronic)^14^. The monomer stopping power *S*_i_ (*E*/60, C_1_) was derived from SRIM 2013^15^. At *E* > 20 MeV, *S*_e_ is more than 10 times greater than *S*_n_, and the former increases with the energy while the latter decreases. Canut et al.^7^ plotted the squared radius of the ion tracks (*R*^2^) versus *S*_e_, and confirmed the square root law:

2a $$R = C(S_{{\text{e}}} - S_{{{\text{e}},{\text{th}}}} )^{{{1}/{2}}} ,$$

where *S*_e,th_ and *C* denote the threshold stopping of ~ 30 keV/nm and a prefactor constant, respectively. The square root law (2a) is a phenomenological law and has a following relation with the analytical thermal spike (ATS) model^16^: The ATS model gives

3a $$R^{{2}} = a\left( 0 \right)^{{2}} {\text{ln}}\left( {S_{{\text{e}}} /S_{{{\text{e}},{\text{th}}}} } \right),\quad \left( {S_{{\text{e}}} < { 2}.{7}S_{{{\text{e}},{\text{th}}}} } \right),$$

3b $$R^{{2}} = \left\{ {{\text{a}}\left( 0 \right)/{2}.{\text{7S}}_{{{\text{e}},{\text{th}}}} } \right\}S_{{\text{e}}}. \left( {S_{{\text{e}}} > { 2}.{7}S_{{{\text{e}},{\text{th}}}} } \right)$$

where *S*_e,th_ and *a*(0) denote the threshold *S*_e_ for the track formation and the initial width of the radial distribution of temperature in the track, respectively. When (*S*_e_ / *S*_e,th_) = (1 + x), where ${\text{x}} \ll 1$, the relation ln (1 + x) ~ x is hold. Then, ln (*S*_e_/*S*_e,th_) is approximated by (*S*_e_/*S*_e,th_ – 1), the square root law (2a) is derived:

4 $$R^{{2}} = a\left( 0 \right)^{{2}} (S_{{\text{e}}} - S_{{{\text{e}},{\text{th}}}} )/S_{{{\text{e}},{\text{th}}}} .$$

However, because of different *S*_e_ dependence between the square root law and the ATS, the deviation between them becomes larger with increasing *S*_e_ as shown in Fig. S-1 in the supplementary materials.

The validity of the square root law was confirmed by Kamarou, et al. in various semiconductors including InP, GaAs, Ge, and Si, irradiated with high energy C_60_ ions^17^. Following the tradition, the square root law is applied in this paper.

In the above-cited studies, the stopping power was calculated using, e.g., TRIM 97^18^. In this study, the previous stopping power values were recalculated using the latest code SRIM 2013^15^. The *S*_e,th_ of 30 keV/nm is extrapolated using SRIM 2013. However, different values of *S*_e,th_ of 37 keV/nm^17^ and 32 keV/nm^7^ were calculated from the same experimental data using various TRIM codes.

As shown in Fig. 1a, the fitting by Eq. (2a) well matched the data points. Note that the abscissa is not plotted with *S*_e_ but with the energy. Following the extrapolation, the track radius becomes null at ~ 17 MeV^7^; that is, no tracks were formed below ~ 17 MeV. While the square root law (2a) could be a rough approximation, the existence of the *S*_e_ threshold is a typical behavior of latent ion tracks formed in materials by SHI irradiation, which is ascribed to the melting heat in the inelastic thermal spike (i-TS) model^19^.

Figure 1a shows that ion tracks of ~ 10 nm in diameter were observed under 6 MeV C_60_ irradiation, far below the hitherto-reported threshold of ~ 17 MeV^7^ (hereafter termed the ‘electronic threshold’). It should be noted that the track diameters at 6 MeV were comparable to those at 40 MeV, and larger than those at 30 MeV. These facts indicate that there is a mechanism for energy deposition not only from *S*_e_ but probably also from *S*_n_, since *S*_e_ monotonously decreased but *S*_n_ increased with decreasing energy as shown in Fig. 1b.

**Figure 2:**
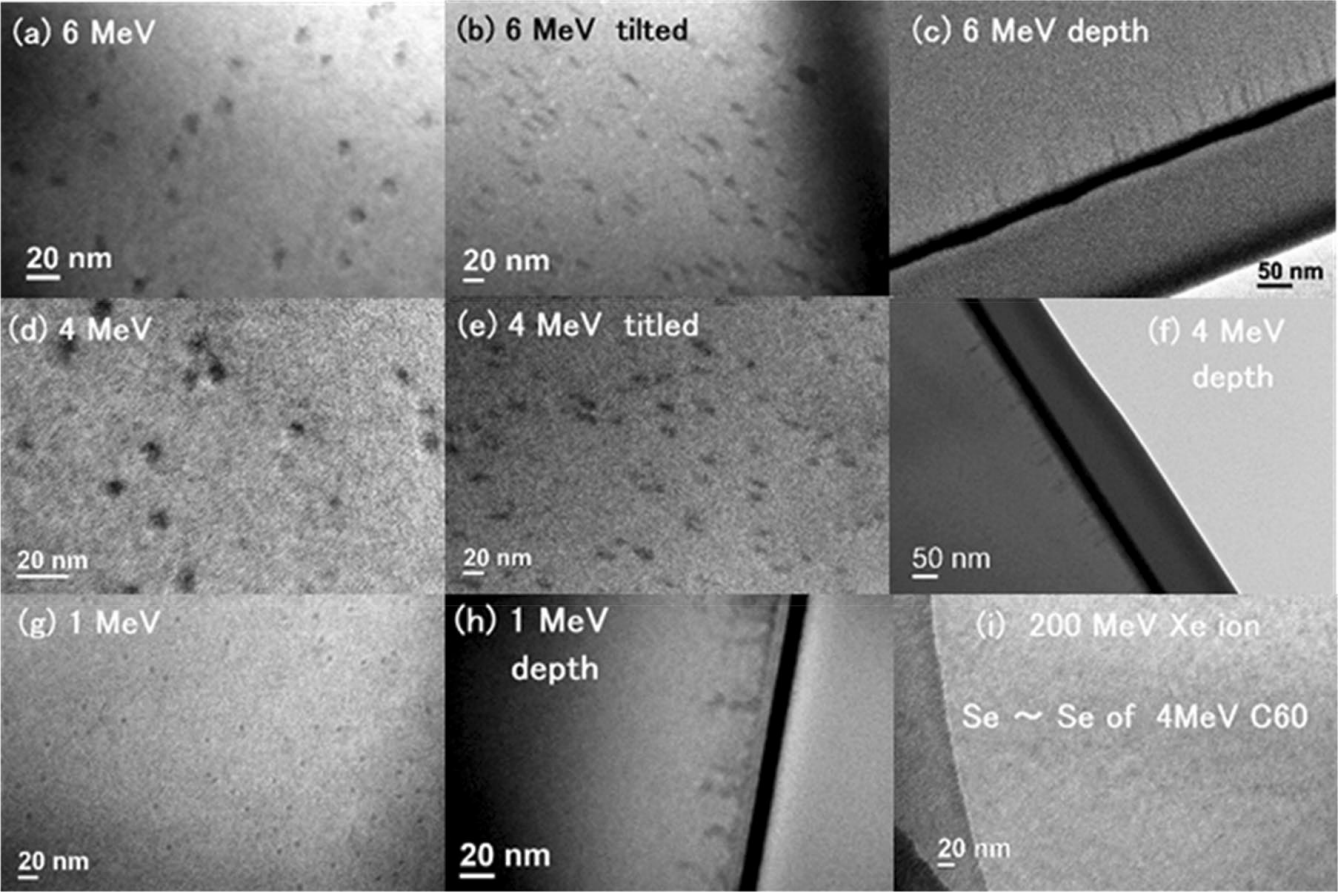
Bright-field TEM images of c-Si samples irradiated with (**a**)–(**c**) 6 MeV C_60_^+^, (**d**)–(**f**) 4 MeV C_60_^+^, (**g**,**h**) 1 MeV C_60_^+^, and (**i**) 200 MeV Xe^14+^ ions. All the samples were prepared by the pre-thinned configurations, except (**c**), (**f**), and (**h**) by the post-thinned configurations. Although both 200 MeV Xe^14+^ ions and 4 MeV C_60_ ions provide almost the same *S*_e_ in c-Si, tracks were not observed in the former case, as shown in (**i**). While 4 and 6 MeV C_60_^+^ ions exhibit almost straight tracks (**c**,**f**), 1 MeV C_60_^+^ ions exhibit skewed tracks, which could indicate crossover from ion tracks to collision cascades.

Figure 2 shows bright-field transmission electron microscopy (TEM) images of irradiated c-Si samples. As described in the Methods section, we prepared TEM specimens in two different configurations, i.e., pre-thinned (planar) and post-thinned (cross-sectional). Figure 2a shows a planar image (i.e., a pre-thinned sample) of c-Si irradiated with 6 MeV C_60_^+^ ions. Dark dots of ~ 10 nm in diameter are observed. The sample was irradiated with an incident angle of 7° to avoid channeling. Since cos (7°) ~ 0.993, the deformed circular images of the tracks could not be due to the non-normal incidence. In all the observed pre-thinned samples, the areal density of the tracks was in the same order of the magnitude as the ion fluence. Figure 2b shows a 30°-tilted image of the same sample (in a lower magnification). The nearly circular dots in Fig. 2a turned to cylinders in Fig. 2b, supporting the fact that the dark regions are ion tracks.

Figure 2c shows a cross-sectional (post-thinned) image of a sample irradiated with 6 MeV C_60_^+^ ions, which indicates the depth profiles of the ion tracks. The black layer is a deposited Pt layer for a surface marker. Many tracks were observed but they were almost free from overlaps. The three different configurations of observations (planar, inclined planar, and cross-section) firmly confirm the formation of the ion tracks. The track lengths showed some variation with the mean length of 66.3 nm and the standard deviation of 7.9 nm. While many hillocks were observed in quartz (SiO_2_) crystal irradiated with the same conditions^20^, the hillocks were not observed in the case of c-Si.

Furuno et al. reported that the tracks could be formed in low-quality evaporated films of Si under 207 MeV Au^13+^ irradiation (*S*_e_ = 17 keV/nm)^21^. Since this report^21^ was inconsistent with other observations in crystalline Si^4,5^, the track formation was ascribed to the low-quality (low thermal conductivity, high electron–phonon coupling, etc.) of the evaporated Si films (probably in an amorphous phase). However, our observation of the tracks cannot be attributed to bad quality of the samples. Our samples were commercial available crystalline Si. Possibly, the pre-thinned samples (e.g., Fig. 2a) could be slightly damaged since they were thinned by focused ion beam (FIB) of 30 keV Ga^+^ before C_60_ irradiation. However, the same tracks are also observed in the post-thinned samples (Fig. 2c), which were not damaged with FIB before C_60_ irradiation. This fact confirmed that the damage in the pre-thinned samples were minimum and probably negligible. Furthermore, the track formation under 1–6 MeV C_60_ irradiation, which we observed, is an intrinsic phenomenon, not induced by pre-damage.

Figure 2d–f show the tracks formed by 4 MeV C_60_^+^ ion irradiation. Compared to the tracks formed by 6 MeV ions, these tracks (4 MeV) were slightly smaller and shorter. Track formation in c-Si was also confirmed with C_60_^+^ ions of 3, 2, and 1 MeV. Figure 2g,h show the track formation with 1 MeV C_60_^+^ ion irradiation, in the pre-thinned (g) and post-thinned (h) configurations, respectively. With decreasing the ion energy to 1 MeV, both the diameter and length of the tracks decreased compared to higher energies.

It should be noted that the shapes of the ion tracks formed by 1 MeV ions are not straight as those formed by higher energies. As well known, the high electronic energy deposition *S*_e_ forms damage regions of straight cylinders, while the high nuclear energy deposition *S*_n_ forms more random and extended collision cascades. As shown in Fig. 1b, a 1 MeV C_60_ ion has comparable high *S*_e_ and high *S*_n_. The formed tracks could be skewed tracks as observed in Fig. 2h, as the consequence of both the contributions of *S*_e_ and *S*_n_.

Figure 2i shows a pre-thinned sample irradiated with 200 MeV Xe^14+^ ions (*S*_e_ = 14 keV/nm and *S*_n_ = 0.05 keV/nm); the *S*_e_ value being comparable to that of the 4 MeV C_60_ ions (*S*_e_ = 15 keV/nm and *S*_n_ = 3 keV/nm). However, no tracks were observed in this case, indicating the importance of the non-negligible *S*_n_ contribution (i.e., the synergy effect) for the track formation.

## Discussion

**Figure 3:**
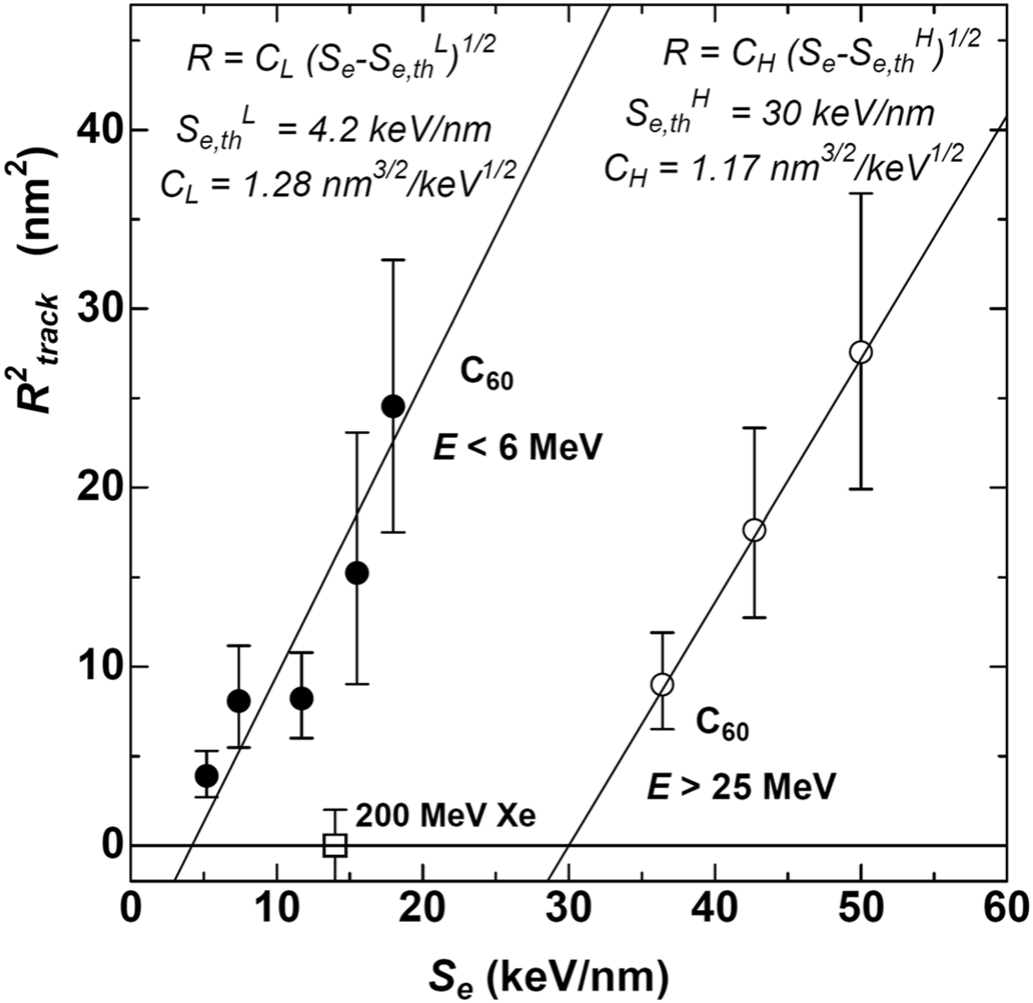
The energy dependence data of the track radii shown in Fig. 1a were plotted with the squared radius *R*^2^ versus electronic stopping power *S*_e_. The solid liens indicate the square root laws as shown by Eq. (2b). The high (open circles) and low (closed circles) energy data fall on the same law with different parameters. The thresholds and the prefactors fitted at high and low energy regions were denoted by *S*_e,th_^H^, *C*_H_, and *S*_e,th_^L^, *C*_L_, respectively. A data point of 200 MeV Xe ion is also indicated.

The data shown in Fig. 1a, i.e., the energy dependence of the track diameters, were plotted with squared radius *R*^2^ versus the electronic stopping power *S*_e_ in Fig. 3. The *S*_e_ was derived from Eq. (1). Both the high energy data (*E* ≥ 25 MeV) and the low energy one (*E* ≤ 6 MeV) are well fitted with the square root laws but different parameter values, i.e.,

2b $$R = C_{i} ({\text{S}}_{{\text{e}}} - {\text{S}}_{{\text{e th}}}^{{\text{i}}} )^{{{1}/{2}}} , \quad \left( {i = L\;{\text{or}}\;H} \right).$$

where the superscripts *L* and *H* denote the low and high energy region, respectively. The threshold stoppings were *S*_e,th_^L^ = 4.2 keV/nm and *S*_e,th_^H^ = 30 keV/nm for *E* ≤ 6 MeV and *E* ≥ 25 MeV, respectively. The high energy threshold was ten times higher than the low energy one. Particularly, the threshold of 4.2 keV/nm was extraordinarily low. Itoh et al. have collected the *S*_e_ thresholds of 43 materials in their paper^22^. Among the 43 materials, those having the thresholds less than 4.2 keV/nm (our case at *E* ≤ 6 MeV) are limited to only four materials: SiO_2_ (2 keV/nm), a-Ge (3 keV/nm), LiF (4 keV/nm), and BaFe_12_O_19_ (4 keV/nm)^22^. Since c-Si is known to have very high threshold, something unusual could happen under the C_60_ ion irradiation at *E* ≤ 6 MeV.

Contrary, the prefactor for the low energy (*C*_L_ = 1.28 nm^3/2^/keV^1/2^ for *E* ≤ 6 MeV) and that for the high energy (*C*_H_ = 1.17 nm^3/2^/keV^1/2^ for *E* ≥ 25 MeV) are almost the same, irrespective of much different energy regions. According to the i-TS model, the threshold *S*_e,th_ relates to the heat for the melting. The similar values of the prefactor *C* for high and low energy regions indicate that the track radius increases in almost the same manner with the additional *S*_e_, i.e., (*S*_e_ – *S*_e,th_), irrespective of *S*_e,th_. Almost the same value of *C*_L_ and *C*_H_ could be a reasonable consequence, because the material is the same, i.e., c-Si. On the other hand, the large difference in the threshold *S*_e,th_ indicates that the melting (or the boiling) is induced with very low value of *S*_e_ in the low energy region, where *S*_n_ is not negligible. Therefore, there must be an additional heat source except *S*_e_. The strongly reduced threshold could be ascribed to much more efficient heat source rather than the *S*_e_ but probably *S*_n_. For reference, a data point of 200 MeV Xe ion is also shown in Fig. 3, which has similar *S*_e_ with 4 meV C_60_ ion but much lower *S*_n_. Clearly the tracks are not formed under 200 MeV Xe irradiation.

Since the tracks formed by the high energy (*E* ≥ 25 MeV) are ascribed to the electronic energy deposition, one might consider that the tracks by the low energy (*E* ≤ 6 MeV) could be formed by the purely nuclear energy deposition, e.g., the collision cascades. However, the low energy tracks (*E* ≤ 6 MeV) also depend on *S*_e_ via the square root law as shown in Fig. 3, indicating that *S*_e_ plays an important role even in the low energy region (*E* ≤ 6 MeV).

**Figure 4:**
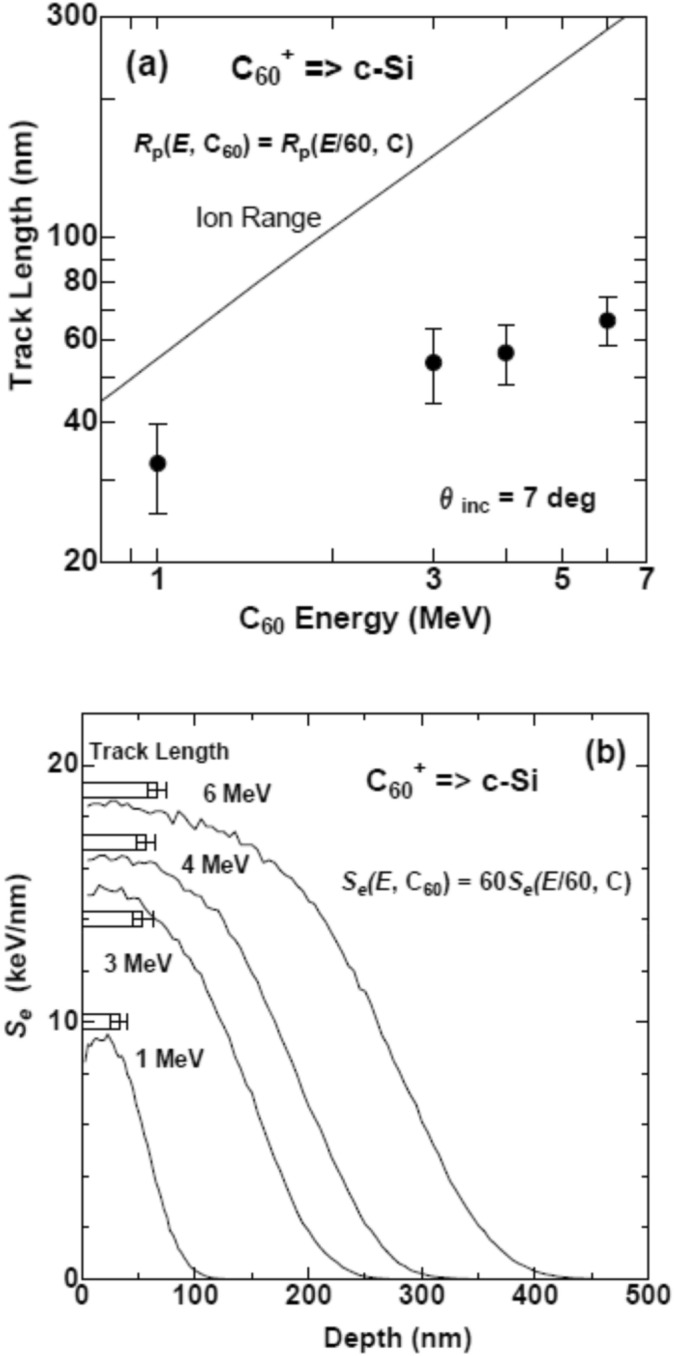
(**a**) Ion energy dependence of the track length of C_60_ ions in c-Si is shown by closed circles. The line is the ion range estimated from the Eq. (5). (**b**) Depth dependence of the electronic energy loss *S*_e_ for *E* = 1, 3, 4, and 6 MeV, calculated by Eq. (1) using SRIM 2013^15^ (www.srim.org). The track lengths observed by XTEM are shown by rectangles with error bars.

Figure 4a shows the track lengths determined from the post-thinned samples by closed circles. Here we need to recall the definition of two quantities: Ion range (projected range) is the depth where implanted ion terminates, while the track length is the length of the modified region formed by an ion. Even beyond the track length, the ion goes further without forming track. The ion range is longer than the track length. The track termination of swift *monomer* ion is induced when the *S*_e_ of the ion decreases below the threshold. Contrary, in the case of C_60_ ions, there is another channel for the track termination, i.e., the fragmentation of the C_60_ ions^8^. Also there are two mechanisms for the fragmentation: (i) Coulomb repulsion between ionized carbon atoms constituting a C_60_ molecule^8^ and (ii) collisions of carbon atoms constituting a C_60_ molecule with Si matrix^8^. Dunlop et al. concluded that the latter (C-Si collisions) are the dominant process for the fragmentation of 30 MeV C_60_ ion irradiation^8^. Since the latter process become more dominant for lower energy, the fragmentation processes in our experiments (*E* = 1–6 MeV) are mostly governed by the C_60_-Si collisions.

If a C_60_ molecule is assumed as 60 independent single C ions with the same energies of *E*/60, the ion range *R*_P_ of a C_60_ ion can be approximated by the ion range of the single C ion with the same velocity, i.e.,

5 $$R_{{\text{P}}} (E,{\text{C}}_{{{6}0}} ) \, = R_{{\text{P}}} (E/60,{\text{C}}_{{1}} ).$$

The right-hand side of the Eq. (5), *R*_P_ (*E/60*, C_1_), i.e., the projected range of single carbon ion in c-Si with the energy of *E*/60, was calculated by SRIM 2013^15^. The calculated ion ranges were plotted in Fig. 4a, which were much longer than the ion tracks observed by TEM.

Figure 4b shows the depth profiles of the electronic energy deposition *S*_e_ induced by a C_60_ ion, which were calculated by Eq. (1). The track lengths are indicated by rectangles with error bars. The incident *S*_e_ values of 6 MeV C_60_ ion at the surface of c-Si is 18.4 keV/nm, which decreases to 18.2 keV/nm at the depth of 66 nm where the track terminated. The incident *S*_e_ value of 4 MeV C_60_ at the surface is 16.3 keV/nm, which is much lower than the *S*_e_ value at the end of the track of 6 MeV C_60_ ion (18.2 keV/nm). Even though, tracks of 57 nm long in average are formed under 4 MeV C_60_ irradiation. In the case of 1 MeV C_60_ irradiation, the incident *S*_e_ at the surface is 8.5 keV/nm, which is less than a half of the *S*_e_ value at the end of the 6 MeV C_60_ track (18.2 keV/nm). However, tracks of 33 nm long in average are formed. From these observations, it is concluded that the termination of the tracks is not induced when the *S*_e_ value becomes below the threshold value but probably when the fragmentation of the C_60_ cluster is induced. When a C_60_ ion is divided into *N* fragments, each fragment has *S*_e_/*N* in average, which results in drastic reduction of the *S*_e_.

**Figure 5:**
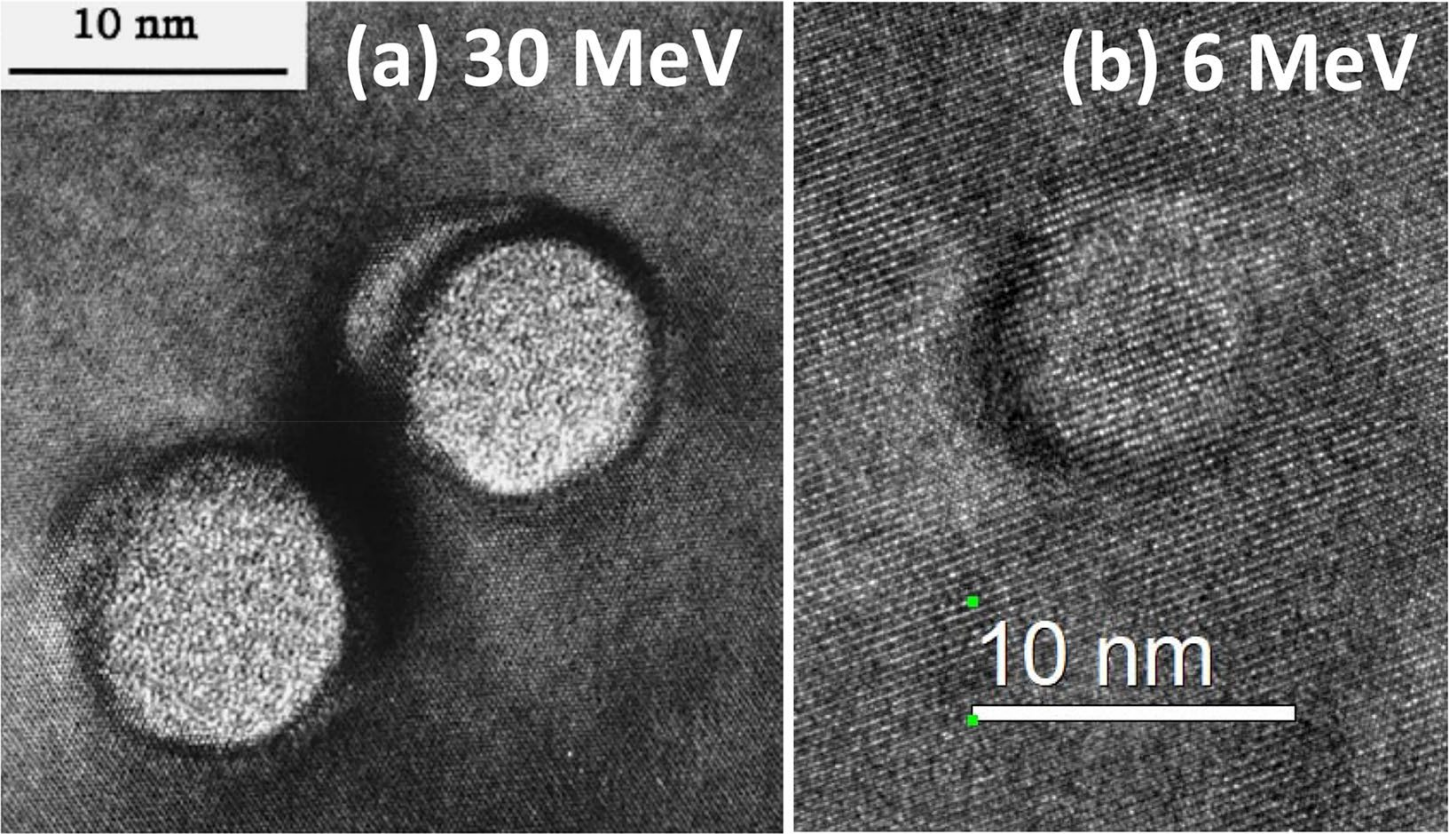
High resolution TEM images of ion tracks in c-Si irradiated with C_60_^+^ ions of (**a**) 30 MeV ( reproduced with permission from A. Dunlop et al. Ref.^8^) and of (**b**) 6 MeV (this work).

Figure 5 exhibits high-resolution (HR) planar TEM images of ion tracks in c-Si irradiated with C_60_ ions of (a) 30 MeV^8^ and (b) 6 MeV. While a nearly circular track was observed in Fig. 5b, a striking observation was that the track region also exhibited lattice fringes, coincided with the unirradiated region, i.e., a defective crystalline track. Dunlop et al. observed amorphous tracks in c-Si irradiated with 30 MeV C_60_ irradiation as shown in Fig. 5a.^8^ While we have also carried out HR-TEM observations of tracks formed by 4 MeV C_60_ ions (not shown) in addition to 6 MeV one, they also showed similar defective crystalline tracks.

Dunlop et al. observed that recrystallization of ion tracks in c-Si during the HR-TEM observation^8^. Since they irradiated c-Si with 30 MeV C_60_ ions, the samples could be much more damaged than our samples irradiated with 6 MeV C_60_ ions. Much quicker recrystallization could be induced in our cases.

Another possibility is due to much shorter length of our tracks: The mean track length was 66.3 nm for 6 MeV C_60_ ions, which is thinner than the TEM sample (~ 100 nm thick). Since the tracks terminate inside the TEM samples, the remaining crystalline parts deeper than the track-length would contribute for the fringes. However, the HR-TEM images are so clear that this explanation seems less plausible. Up to now, a lot of studies have been carried out to discover ion tracks in c-Si using SHIs^4,5^. However, there are no reports on defective crystalline tracks in c-Si.

**Figure 6:**
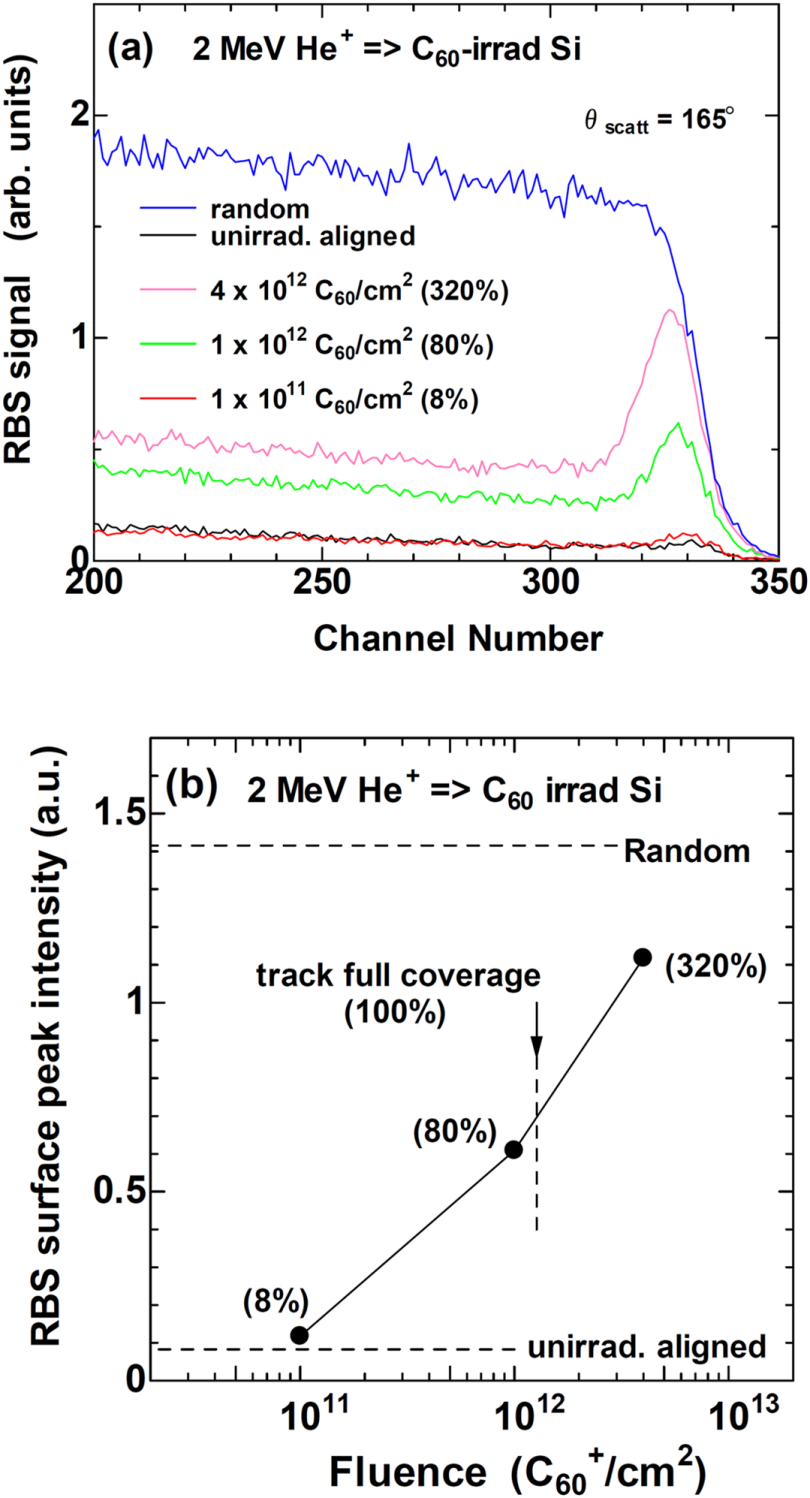
(**a**) RBS/C spectra of c-Si in unirradiated state and irradiated with 6 MeV C_60_^+^ ions to three different fluences. (**b**) Fluence dependence of the surface peak around 330 channel. Horizontal broken lines indicate the random and aligned levels in unirradiated sample. Numbers in parentheses indicate the track coverage ratio at the fluences, which is defined by eq. (6). The vertical broken line indicates the track full coverage, i.e., 100%.

To confirm the defective crystalline tracks, Rutherford backscattering spectrometry and channeling (RBS/C) measurements were carried out. Figure 6a exhibits RBS/C spectra of random and aligned configurations of an unirradiated sample, and of the aligned configurations of the samples irradiated with 6 MeV C_60_^+^ ions at three different fluences. With increasing the fluence, the surface peak around 330 channel, which is due to the scattering by the tracks, increased but does not reached at the random level. The numbers in parentheses indicate the track coverage ratio (TCR) at the surface, which is defined as

6 $${\text{TCR }} = \pi R^{{2}} \Phi$$

where *R* and Φ denote the mean radius of the ion track and the C_60_ fluence, respectively.

The intensity of the surface peak was plotted with the fluence in Fig. 6b. From the track radius shown in Fig. 1a, TCR reaches at 100% at the fluence of 1.3 × 10^12^ C_60_/cm^2^, where almost all the surface of the sample is covered by the tracks. If the tracks could be amorphous, the surface peak intensity would reach the random level and would saturate at TCR ~ 100%. However, the experiments showed that the surface peak intensity reached at only ~ 46% of the random level at TCR of 100% and that the intensity monotonically increased even beyond the TCR of 100%. It should be noted that even ~ 46% of the random level could indicate significant disorder, defects or possibly partial amorphization. However, HR-TEM image (Fig. 5b) does not show any evidence of partial amorphization.

Furthermore, the surface peak increased to the fluence to 4 × 10^12^ C_60_/cm^2^ (TCR = 320%), where the multiple overlaps of the tracks are expected. Even though, the surface peak intensity did not reach at the random level of the RBS/C measurements. Consequently, these observations support the formation of defective crystalline ion tracks, rather than amorphous ones, under 6 MeV C_60_ ion irradiation.

At the beginning, we considered that the tracks observed in Fig. 5b were originally amorphous but crystallized by electron beam irradiation under HR-TEM observation. However, the samples used for the RBS/C measurements were not irradiated by electron beam but only 2 MeV He^+^ ions. Consequently, the crystallinity of the ion tracks has been confirmed by two different methods, i.e., HR-TEM and RBS/C, which further supports that the defective crystalline tracks in c-Si are not a consequence of improper experimental procedures but an intrinsic nature of this system.

Szenes and Toth^23^ irradiated yttrium-iron-garnet (YIG) Y_3_Fe_5_O_12_ with C_60_ ions of 3.5, 5, and 7 MeV, and observed the ion track formation. To evaluate the synergy effect of *S*_e_ and *S*_n_ on track formation, they applied an effective stopping power defined by

7 $$S_{{{\text{eff}}}} = S_{{\text{e}}} + K \, S_{{\text{n}}} ,$$

with a coefficient *K* of 2.2 for the YIG. Kitayama et al.^24^ irradiated amorphous SiN films with 540 keV C_60_^2+^ ions, and evaluated the synergy effect on the electronic and collisional sputtering. To explain the data, they used *K* = 2.5. We have adopted this formalism. As shown in Fig. 1b, even assuming *K* = 3, *S*_eff_ is less than the electronic threshold of 30 keV/nm at 6 MeV. If *K* = 5 is assumed, *S*_eff_ becomes greater than the threshold of 30 keV/nm at 6 MeV, i.e., the track formation is approved. In this case, however, *S*_eff_ at 1 MeV is higher than at 6 MeV, which is inconsistent with the larger track diameters at 6 MeV than those at 1 MeV. This is because Eq. (7) is an empirical rule, which holds in limited energy region only. If it would hold also in very low energy where *S*_e_ <  < *S*_n_, the effective stopping power *S*_eff_ could inconsistently be ~ *KS*_n_, which should be higher than *S*_n_ since *K* > 1. A transition from 6 to 1 MeV could happen because the track shapes at 6 MeV are nearly straight (Fig. 2c) but those at 1 MeV are skewed cylinder shapes (Fig. 2h). Different spatial distributions of *S*_e_ (straight cylinders) and *S*_n_ (skewed cylinder) could reduce the synergy effect efficiency, which has already been reported in zircon^25^.

While the mechanism of the track formation at 1–6 MeV is still an open question, we suggest two issues: (i) As pointed by Weber et al.^9^, the track radii became larger in pre-damaged samples. For C_60_ ions of 1–6 MeV, *S*_n_ is not negligible to *S*_e_. The damage formed by *S*_n_ assists the track formation by *S*_e_. Since the fluence is low, the track formation by *S*_e_ assisted by *S*_n_ is induced by the same ion. This is not the pre-damage effect but the “in-situ” damage effect. Another issue is that Chettah et al.^13^ proposed the boiling transition for the track formation at 30–40 MeV C_60_ ions from the analysis of the inelastic thermal spike model. We speculate that the tracks by the melting transition could not been observed because of the perfect recrystallization under SHI irradiation where *S*_n_ is negligible. We attribute the track formation under 30-40 MeV C_60_ irradiation to the boiling transition. Contrary, non-negligible *S*_n_ under 1–6 MeV C_60_ irradiation prevents the perfect recrystallization at the melting transition, which results in the track formation by the melting transition.

## Summary

The ion energy dependence of track formation in c-Si, and the relevant stopping powers, are schematically summarized in Fig. 1c. The C_60_ irradiation higher than 25 MeV, *S*_n_ is much less than *S*_e_. Consequently the synergy effect is not expected (i.e., *S*_e_ > *S*_e,th_ >  > *S*_n_). The damage formation is governed only by *S*_e_. When *S*_e_ decreases below the threshold, i.e., *S*_e_ < *S*_e,th_, tracks are expected to be no longer formed. (This is only due to the extrapolation). However, a further decrease in the energy results in an increase in *S*_n_, activating the synergy effect: Tracks are formed once again at 1–6 MeV. With further decreasing energy, the contribution of *S*_n_ increases further, finally resulting in cascade-like tracks being observed at 1 MeV.

While the *S*_e,th_ determined from the square root law at *E* ≥ 25 MeV was reasonably high as 30 keV/nm, the *S*_e,th_ at *E* ≤ 6 MeV was 4.2 keV/nm, which is extraordinarily low and inconsistent with the fact that c-Si is insensitive to SHIs. There must be another heat source other than *S*_e_, but probably *S*_n_ for C_60_ irradiations at *E* ≤ 6 MeV. However, it should be noted that the track diameters at low energy (*E* ≤ 6 MeV) depend on *S*_e_ via the square root law. It seems that the *S*_e_ plays an important role in the track formation also at low energy (*E* ≤ 6 MeV). Purely nuclear origin, such as the collision cascades are excluded, while it does not mean to exclude the synergy effects between *S*_e_ and *S*_n_. The track length is determined not by a decrease of *S*_e_, but by the fragmentation of C_60_ molecules. Another scenario is that the track formations at 6 MeV and those at 30 MeV could be ascribed to the melting and boiling transitions, respectively. Whilst the tracks by the melting transition are fully recrystallized under SHI irradiations, the recrystallization is hindered due to non-negligible nuclear collision contribution.

## Methods

### ***C***_***60***_*** ion irradiation***

The irradiation of C_60_^+^ ions was conducted at the Takasaki Advanced Radiation Research Institute (TARRI), of the National Institutes for Quantum and Radiological Science and Technology (QST), using a 3 MV tandem accelerator and a newly developed high-flux C_60_ negative ion source. The samples were mostly irradiated to a low fluence of 5 × 10^10^ C_60_/cm^2^ to avoid overlaps between the tracks. For precise control of the low fluence, the ion flux was reduced to below 50 pA through an aperture of 3 mm in diameter, while using the high-flux ion source. The incident angle was set to 7° to avoid any channeling effect. Since cos (7°) = 0.993, beams deviating from the normal incidence did not significantly modify the shapes of the tracks. For comparison, some samples were irradiated with 200 MeV Xe^14+^ ions from the tandem accelerator in the Japan Atomic energy Agency (JAEA), Tokai Research and Development Center.

### Sample preparation

Silicon samples were cut from commercially available Si wafers of p-type conduction (boron-doped), with resistivity of ~ 1 Ωcm, surface orientation of < 1 1 1 > , and thickness of ~ 0.38 mm. The samples were mechanically cut into 3 mm × 4 mm rectangles, which are hereafter called bulk samples. The bulk samples were immersed in hydrofluoric acid to remove the surface oxide.

Two different TEM specimens (pre-thinned and post-thinned) were prepared from the bulk samples. In the case of the post-thinned samples, a group of bulk samples were irradiated by a C_60_^+^ beam with an incident angle of 7° to the 3 mm × 4 mm faces, and the TEM specimens were milled down to a thickness of ~ 100 nm to observe the depth profile of the ion tracks. The TEM specimens were fabricated with 30 keV Ga focused ion beam (FIB) milling. To identify the surface position in the cross-sectional configuration, a thin layer of Pt was deposited on the sample surface before the FIB milling. The other bulk samples (pre-thinned) were first milled down to a thickness of ~ 100 nm, and then irradiated with C_60_^+^ ions with an incident angle of 7° to observe the track faces. See Fig. S-2 for details.

### TEM observation

TEM observation of both configurations (i.e., the depth and planar profiles) was conducted using a JEOL JEM-2100 microscope (with a LaB_6_ thermal emission e-gun). HRTEM observation was carried out using JEOL JEM-2100F microscope (with a Schottky-type field emission e-gun). Both the acceleration voltages were 200 kV. According to past literature, the tracks in c-Si were recrystallized with prolonged TEM observation. Careful observations were performed to minimize the electron beam current and TEM observation time.

### Rutherford backscattering spectrometry and channeling (RBS/C) measurements

He^+^ ions of 2 MeV from a single-end accelerator in QST-Takasaki were used. The beam size was 1 mm in diameter and the scattering angle was 165°. Scattered ions were monitored by a surface-barrier detector, with the accumulated charge of 10 μC for a spectrum.

## Supplementary Information

Supplementary Information.

## Data Availability

The datasets and materials generated during the current study are available from the corresponding author on reasonable request.
