# Supplementary material for: Ion tracks in silicon formed by much lower energy deposition than the track formation threshold
Source: Sci Rep. 2021 Jan 8;11:185. doi: 10.1038/s41598-020-80360-8 (PMC7794553; doi:10.1038/s41598-020-80360-8)
Supplement: Supplementary file 1 — Supplementary Information. [file 41598_2020_80360_MOESM1_ESM.pdf]

Supplementary Materials

Ion tracks in silicon formed by much lower energy deposition than the track formation threshold

H. Amekura [1)*], M. Toulemonde [2)], K. Narumi [3)], R. Li [1),4)], A. Chiba [3)], Y. Hirano [3)], K. Yamada [3)], S. Yamamoto [3)], N. Ishikawa [5)], N. Okubo [5)], and Y. Saitoh [3)]

[1)] *National Institute for Materials Science (NIMS), Tsukuba, Japan*

[2)] *CIMAP, Caen, France*

[3)] *National Institutes for Quantum and Radiological Science and Technology (QST), Takasaki, Japan,*

[4)] *Shandong University, Jinan, China*

[5)] *Japan Atomic Energy Agency, Tokai, Japan*

*Corresponding author: amekura.hiroshi@nims.go.jp

S1. The relation between the square root law and the analytical thermal spike (ATS) model

The analytical thermal spike (ATS) model describes the electronic stopping power S_e

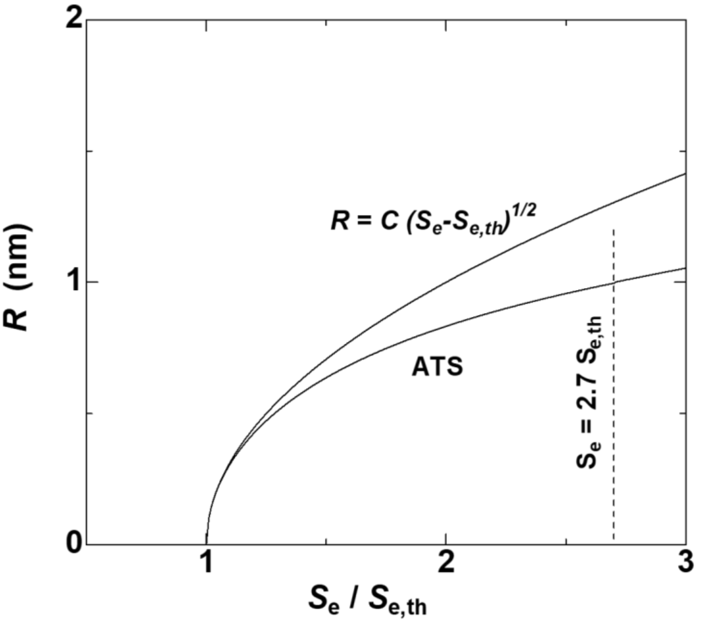

Fig. S-1. Electronic energy stopping S_e dependence of track radius calculated by two models: the analytical thermal spike (ATS) model and the square root law, $R = C\ (S_e - S_{e,th})^{1/2}$. $S_{e,th}$ denotes the threshold S_e for the track formation.

dependence of the track radius R following numerical forms:

$$R^2 = a(0)^2 \ln(S_e/S_{e,th}), \qquad (S_e < 2.7S_{e,th}), \qquad (3a)$$

$$R^2 = \{a(0)/2.7S_{e,th}\}\ S_e. \qquad (S_e > 2.7S_{e,th}) \qquad (3b)$$

where $S_{e,th}$ and $a(0)$ denote the threshold S_e for the track formation and the initial width of the radial distribution of temperature in the track, respectively. When (1 + x) = (S_e / $S_{e,th}$) ~1, the relation ln (1 + x) ~ x is hold. Then, ln ($S_e/S_{e,th}$) is approximated by ($S_e/S_{e,th}$ – 1), the square root law (2a) is derived:

$$R^2 = a(0)^2\ (S_e - S_{e,th})/\ S_{e,th..} \qquad (4)$$

which is similar to the square root law, i.e.,

$$R = C\ (S_e - S_{e,th})^{1/2} \qquad (2a).$$

However, this coincidence is only valid when $S_e \sim S_{e,th}$. With increasing Se, the deviation between two formulae becomes larger as shown in Fig. S1.

S2. Pre-thinned and post-thinned sample configurations

As briefly described in the method part in the main text, two different configurations of samples were observed by transmission electron microscopy (TEM). Figure S-2 depicts schematic views of both the configurations.

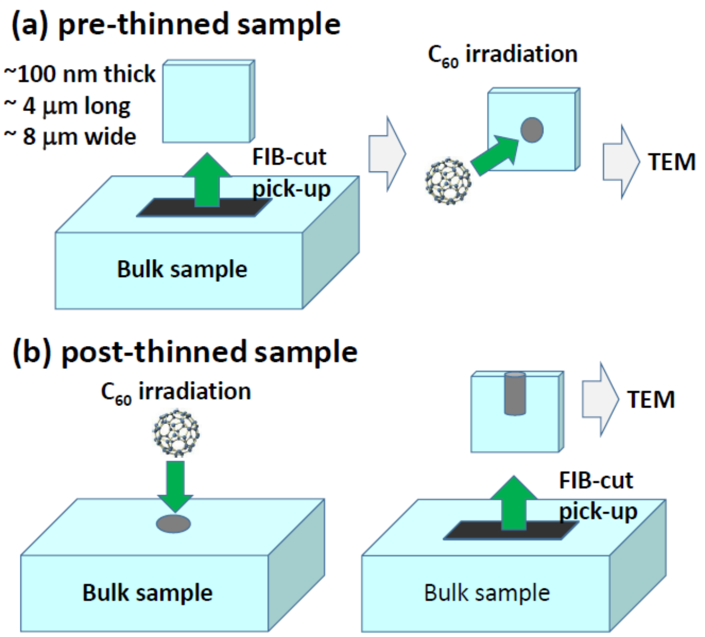

Fig. S-2. Schematically depicted configurations of (a) pre-thinned and (b) post-thinned samples. (a) is used for the observation of track shapes and diameters, while (b) is used for that of track length.
